# Activated CD8^+^CD38^+^ Cells Are Associated With Worse Clinical Outcome in Hospitalized COVID-19 Patients

**DOI:** 10.3389/fimmu.2022.861666

**Published:** 2022-03-14

**Authors:** Anna Bobcakova, Martina Barnova, Robert Vysehradsky, Jela Petriskova, Ivan Kocan, Zuzana Diamant, Milos Jesenak

**Affiliations:** ^1^ Centre for Primary Immunodeficiencies, Clinic of Pneumology and Phthisiology, Jessenius Faculty of Medicine, Comenius University in Bratislava, Martin University Hospital, Martin, Slovakia; ^2^ Department of Clinical Immunology and Allergology, Martin University Hospital, Martin, Slovakia; ^3^ Department of Respiratory Medicine and Allergology, Institute for Clinical Science, Skane University Hospital, Lund University, Lund, Sweden; ^4^ Department of Microbiology Immunology and Transplantation, KU Leuven, Catholic University of Leuven, Leuven, Belgium; ^5^ Department of Respiratory Medicine, First Faculty of Medicine, Charles University and Thomayer Hospital, Prague, Czechia; ^6^ Centre for Primary Immunodeficiencies, Clinic of Pediatrics, Jessenius Faculty of Medicine, Comenius University in Bratislava, Martin University Hospital, Martin, Slovakia

**Keywords:** SARS-CoV-2, COVID-19, immune cell dysregulation, activated CD8^+^ cells, clinical outcome, immunologic predictors

## Abstract

Severe acute respiratory syndrome coronavirus 2 (SARS-CoV-2), that spread around the world during the past 2 years, has infected more than 260 million people worldwide and has imposed an important burden on the healthcare system. Several risk factors associated with unfavorable outcome were identified, including elderly age, selected comorbidities, immune suppression as well as laboratory markers. The role of immune system in the pathophysiology of SARS-CoV-2 infection is indisputable: while an appropriate function of the immune system is important for a rapid clearance of the virus, progression to the severe and critical phases of the disease is related to an exaggerated immune response associated with a cytokine storm. We analyzed differences and longitudinal changes in selected immune parameters in 823 adult COVID-19 patients hospitalized in the Martin University Hospital, Martin, Slovakia. Examined parameters included the differential blood cell counts, various parameters of cellular and humoral immunity (serum concentration of immunoglobulins, C4 and C3), lymphocyte subsets (CD3^+^, CD4^+^, CD8^+^, CD19^+^, NK cells, CD4^+^CD45RO^+^), expression of activation (HLA-DR, CD38) and inhibition markers (CD159/NKG2A). Besides already known changes in the differential blood cell counts and basic lymphocyte subsets, we found significantly higher proportion of CD8^+^CD38^+^ cells and significantly lower proportion of CD8^+^NKG2A^+^ and NK NKG2A^+^ cells on admission in non-survivors, compared to survivors; recovery in survivors was associated with a significant increase in the expression of HLA-DR and with a significant decrease of the proportion of CD8^+^CD38^+^cells. Furthermore, patients with fatal outcome had significantly lower concentrations of C3 and IgM on admission. However, none of the examined parameters had sufficient sensitivity or specificity to be considered a biomarker of fatal outcome. Understanding the dynamic changes in immune profile of COVID-19 patients may help us to better understand the pathophysiology of the disease, potentially improve management of hospitalized patients and enable proper timing and selection of immunomodulator drugs.

## Introduction

Since late 2019, COVID-19 pandemic has spread all around the world, causing over 5,5 million deaths (1). Despite extensive vaccination efforts, the limited vaccine supply in low-income countries, the vaccine hesitancy, the emergence of new virus variants and the waning of postvaccination protection leave the world still far from reaching herd immunity (2–4). Consequently, the healthcare system of many countries is seriously overwhelmed by recurrent pandemic waves of the virus.

The clinical spectrum of COVID-19 can range from asymptomatic cases (tested positive for SARS-CoV-2 without clinical symptoms), through mild (various mild symptoms without dyspnea or signs of pneumonia on chest imaging) and moderate cases (signs of pneumonia without the need of oxygen supplementation), to severe (signs of pneumonia with oxygen saturation < 94% on room air, PaO2/FiO2 < 300 mmHg, respiratory rate > 30 breaths/minute, or lung infiltrates affecting more than 50% of the lung parenchyma) and critical cases (respiratory failure, septic shock, multiple organ failure) (**Table 1**) (5). A model of 3 stages of COVID-19 was suggested (6). Stage I represents early infection, that can progress to stage II, *i.e.*, pulmonary stage without (IIa) or with hypoxia (IIb), and in a minority of patients further progressing into the most severe stage (III) associated with systemic hyperinflammation (6).

**Table 1:** Categories of COVID-19 disease course in relation to the severity of the illness.

Category	Characteristic
**asymptomatic**	tested positive for SARS-CoV-2 without clinical symptoms
**mild**	various mild symptoms without dyspnea or signs of pneumonia on chest imaging
**moderate**	signs of pneumonia without the need of oxygen supplementation
**severe**	signs of pneumonia with oxygen saturation < 94% on room air, PaO2/FiO2 < 300 mmHg, respiratory rate > 30 breaths/minute, or lung infiltrates affecting more than 50% of the lung parenchyma
**critical**	respiratory failure, septic shock, multiple organ failure

According to (5).

Although several risk factors are recognized to be associated with severe or critical disease due to SARS-CoV-2 infection, COVID-19 may occasionally threaten the life of previously healthy young people. In general, patients with advanced age, men, those with chronic diseases (especially arterial hypertension, diabetes, obesity, chronic lung disease, heart, liver and kidney diseases, malignant tumors, selected immunodeficiencies) and pregnant women, are more prone to develop severe or critical COVID-19 (7).

Furthermore, a spectrum of biochemical and hematological parameters was suggested as markers of disease progression. Poor clinical outcome was associated with lymphopenia, thrombocytopenia, neutrophilia, elevated neutrophil-to-lymphocyte ratio, elevated D-dimer, CRP, PCT, CK, AST, ALT, creatine and LDH (8, 9). Longitudinal changes in energy metabolism were also described as a factor of disease progression (10).

The immune system plays a crucial role in the pathogenesis and pathophysiology of COVID-19. In early stages, its role is indisputable in the host defense against the virus, while it acts as an important driver of worsening and progression of the disease to the most severe stages. Therefore, early recognition of COVID-19 symptoms as well as the immune response (dysfunction) could be important for proper timing and the choice of adequate treatment (6).

In search of potential biomarkers and to better understand the immunological and pathophysiological mechanisms driving the disease, several authors have analyzed the immune profile of COVID-19 patients (11–18). As a follow-up on our previous observations (11), we analyzed the differences in the immune profile of hospitalized COVID-19 patients in relation to the disease course and the clinical outcome. We focused on longitudinal changes in the expression of activation and inhibitory molecules, including rarely reported NKG2A on CD8^+^ and NK cells.

## Patients and Methods

**Figure 1:**
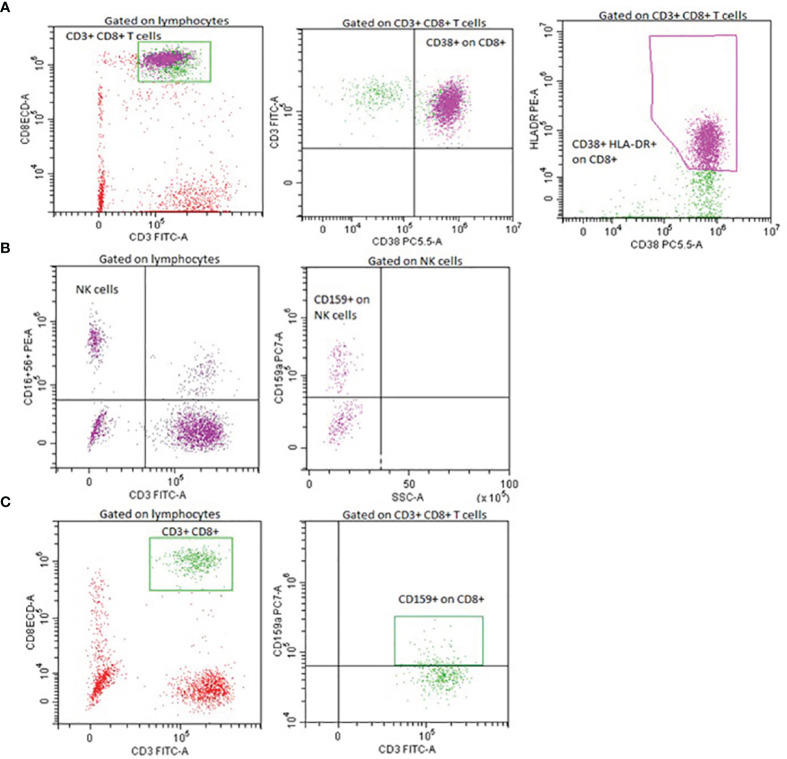
Representative flow cytometry plots of lymphocyte activation and inhibitory markers expression. Expression of CD38 and HLA-DR on CD8^+^ cells **(A)**, expression of NKG2A/CD159 on NK cells **(B)**, expression of NKG2A/CD159 on CD8^+^ cells **(C)**.

This was a single-center observational study. We analyzed the immune profile of 823 adult COVID-19 patients (**Table 2**) hospitalized in the Martin University Hospital, Martin, Slovakia, during the period March 2020 – August 2021. Assessed parameters included differential blood cell counts, serum concentration of immunoglobulins IgG, IgA, IgM, IgE and complement components C3 and C4, flow cytometric phenotyping of lymphocyte subsets (CD3^+^, CD4^+^, CD8^+^, CD19^+^, NK), IRI (immunoregulatory index, CD4^+^/CD8^+^), expression of selected activation markers (CD38 on CD8^+^ cells, HLA-DR on CD3^+^ cells, CD38 and HLA-DR co-expression on CD8^+^ cells) and inhibitory markers (CD159/NKG2A) on CD8^+^ and NK cells) (**Figure 1**).

**Table 2:** Characteristics of patients included in the study and the summary of the therapeutic approaches.

	A (n=103)	B (n=383)	C (n=90)	D (n=206)	E (n=41)
**Sex (male)**	48 (46.7%)	206 (53.8%)	60 (66.7%)	115 (55.8%)	19 (46.3%)
**Age (Mean ± SD)**	63.62 ± 13.95	64.16 ± 14.53	61.16 ± 12.57	75.79 ± 11.36	64.05 ± 19.97
**Chronic ischemic heart disease**	34 (33.0%)	162 (42.3%)	23 (25.6%)	144 (69.9%)	19 (46.3%)
**Hypertension**	65 (63.1%)	270 (70.5%)	63 (70.0%)	179 (86.9%)	25 (61.0%)
**Diabetes**	29 (28.2%)	120 (31.3%)	34 (37.8)	88 (42.7%)	12 (29.3%)
**Obesity**	20 (19.4%)	140 (36.6%)	42 (46.7%)	63 (30.6%)	9 (22.0%)
**Arrhythmia**	15 (14.6%)	70 (18.3%)	6 (6.7%)	62 (30.1%)	14 (34.1%)
**Bronchial asthma**	12 (11.7%)	36 (9.4%)	4 (4.4%)	19 (9.2%)	2 (4.9%)
**Chronic obstructive pulmonary disease**	10 (9.7%)	44 (11.5%)	8 (8.9%)	33 (16.0%)	3 (7.3%)
**Thyroid gland disease**	8 (7.8%)	65 (17.0%)	13 (14.4%)	39 (18.9%)	6 (14.6%)
**Chronic kidney disease**	18 (17.5%)	59 (15.4%)	12 (13.3%)	73 (35.4%)	10 (24.4%)
**Dyslipidemia**	36 (35.0%)	141 (36.8%)	27 (30.0%)	84 (40.8%)	10 (24.4%)
**Stroke history**	9 (8.7%)	27 (7.0%)	9 (10.0%)	38 (18.4%)	5 (12.2%)
**Cancer**	15 (14.6%)	21 (5.5%)	5 (5.6%)	30 (14.6%)	10 (24.4%)
**Hematological malignancy**	2 (1.9%)	6 (1.6%)	2 (2.2%)	12 (5.8%)	1 (2.4%)
**Interstitial lung disease**	1 (0.97%)	6 (1.6%)	1 (1.1%)	7 (3.4%)	2 (4.9%)
**Autoimmunity**	7 (6.8%)	24 (6.3%)	6 (6.7%)	20 (9.7%)	3 (7.3%)
**Immunodeficiency**	1 (0.97%)	3 (0.78%)	5 (5.6%)	3 (1.5%)	0
**Cirrhosis**	0	0	0	4 (1.9%)	2 (4.9%)
**Pregnancy**	0	2 (0.52%)	3 (3.3%)	0	1 (2.4%)
**Venous thromboembolism (during COVID-19)**	7 (6.8%)	20 (5.2%)	5 (5.6%)	7 (3.4%)	0
**Therapeutic approaches**					
**Systemic corticosteroids**	45 (43.7%)	344 (89.8%)	86 (95.6%)	177 (85.9%)	15 (36.6%)
**Antivirals (remdesivir, favipiravir)**	13 (12.6%)	146 (38.1%)	38 (42.2%)	70 (34.0%)	4 (9.8%)
**Baricitinib**	4 (3.9%)	28 (7.3%)	15 (16.7%)	16 (7.8%)	0
**Tocilizumab**	0	1 (0.3%)	4 (4.4%)	3 (1.5%)	0
**Anakinra**	0	1 (0.3%)	2 (2.2%)	0	0

The conventional flow cytometry was used according to the following procedure. The full blood of all patients was collected in collection tubes with EDTA. Fluorescence labeled monoclonal antibodies against selected antigens were added to cell suspensions: tetraCHROME 1 (CD45-FITC/CD56-RD1/CD19-ECD/CD3-PC5), tetraCHROME 2 (CD45-FITC/CD4-RD1/CD8-ECD/CD3-PC5), CD16-PE, CD3-FITC, anti-HLA-DR-PE, CD4-PC5, CD45-FITC, CD45RO-PE, CD8-ECD, CD38-PC5, CD159A-PC7) (Beckman Coulter, CA, USA) and samples were incubated in the dark at optimal laboratory temperature for 30 minutes. Analysis of the cell surface expression was performed using a DxFLEX flow cytometer (Beckman Coulter, CA, USA). Isotype controls with irrelevant specificities were used as negative controls.

All parameters were examined on admission and after one week (except for serum concentration of immunoglobulins and complement, which were measured on admission only). Patients were enrolled continuously without any selection bias, enrolled patients were not aware of previous SARS-CoV-2 infection. The period of the patient enrolment corresponded to the circulation of the wild-type virus, Alpha, Beta, Gamma and Delta variant strains in Europe (19). Wild-type and especially Alpha variants were dominant in Slovakia during the whole period, except for July and August 2021, when Delta variant spread in Slovakia (20).

Patients were divided into 5 groups according to the severity of their COVID-19 (5). Group A (n=103) consisted of patients with mild to moderate COVID-19 course (without the need of oxygen supplementation), group B (n=383) included patients with severe COVID-19 (bilateral pneumonia with hypoxemic respiratory failure), group C (n=90) comprised patients with critical course of COVID-19 (hospitalization in ICU with invasive or non-invasive ventilation support), group D (n=206) consisted of deceased COVID-19 patients and group E (n=41) included patients tested positive for SARS-CoV-2 hospitalized for a different non-respiratory condition. Median time to death from admission to the hospital in group D was 10 days (IQR 5, 15). All patients were followed until recovery and the classification was made with respect to the overall course of hospitalization.

For each parameter, we analyzed differences among groups A – E, differences between survivors hospitalized due to COVID-19 (groups A – C) and non-survivors (group D) and the changes over time in survivors (groups A – C) and non-survivors (group D). We did not include patients of group E in comparisons between survivors and non-survivors due to the heterogeneity within this group and a potentially significant impact of the main comorbidity, which lead to the hospitalization, both on the immune profile as well as on the clinical outcome. In addition, ROC curves and multiple logistic regression analyses were performed to examine if any of the parameters could be considered as (an) independent risk factor(s) for the fatal outcome of COVID-19.

Results were calculated with GraphPad Prism version 9.2.0 for Mac, GraphPad Software, San Diego, California USA, www.graphpad.com. Non-parametric versions of statistical tests were applied (Kruskal-Wallis, Mann-Whitney test, and Wilcoxon rank-sum test).

The study was approved by the local Ethical Committee (Decision No. EK UNM 77/2020, EK JLF UK 74/2021).

## Results

### COVID-19 Non-Survivors Have Significantly Lower Concentration of IgM and C3 on Admission to the Hospital

**Figure 2:**
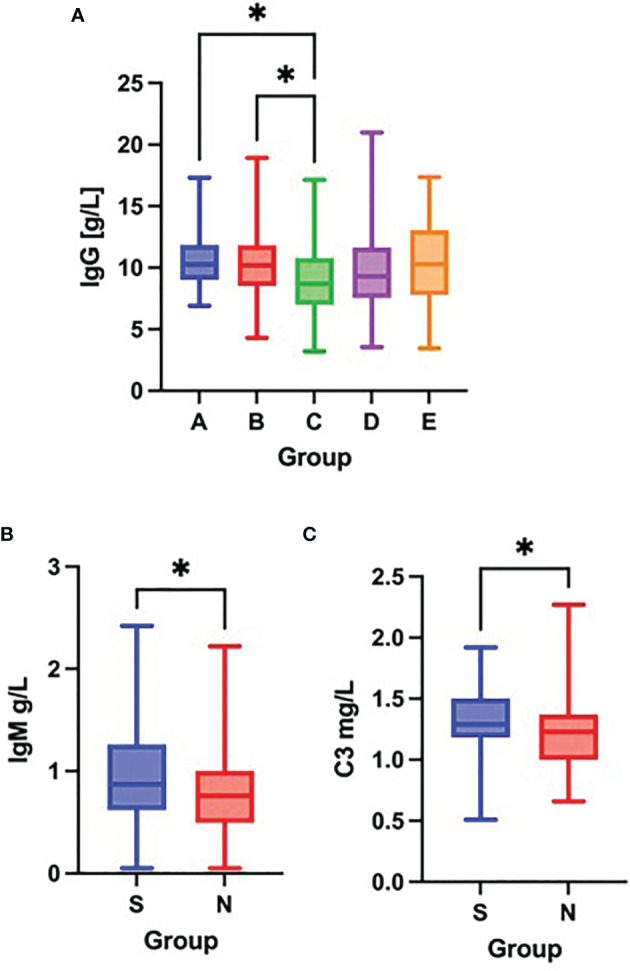
Differences in the serum concentration of IgG **(A)** among groups A – E (Kruskal-Wallis test), differences in the serum concentration of IgM **(B)** and C3 **(C)** between survivors [S] and non-survivors [N] (Mann-Whitney test) on admission to the hospital. *p < 0.05.

As compared to groups A and B, patients in group C had significantly decreased concentration of IgG on admission (**Figure 2A**). No further differences were observed in serum IgG, IgA, IgM, IgE or C3, C4 concentration across the different patient groups on admission. However, comparisons between survivors (groups A – C) and non-survivors (group D) revealed significantly lower concentrations of IgM and C3 in non-survivors on admission. (**Figures 2B, C**).

### Fatal Outcome Is Associated With a Further Decrease in the NK Cell Counts

**Figure 3:**
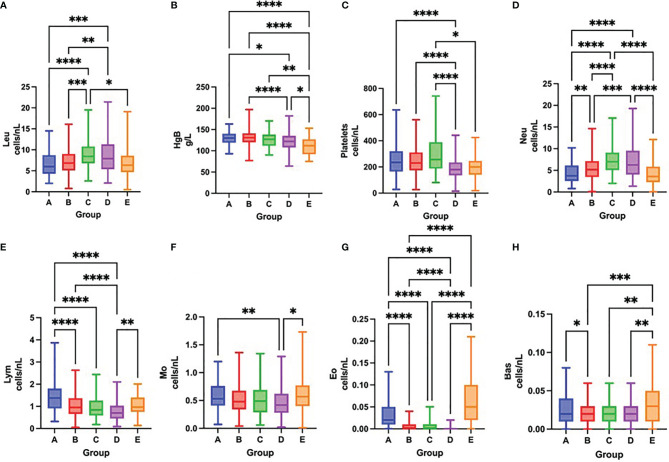
Differences in the total number of leukocytes **(A)**, concentration of hemoglobin **(B)**, total number of platelets **(C)**, neutrophils **(D)**, lymphocytes **(E)**, monocytes **(F)**, eosinophils **(G)** and basophils **(H)** among groups A – E on admission to the hospital, Kruskal-Wallis test. *p < 0.05; **p < 0.01; ***p < 0.001; ****p < 0.0001.

**Figure 4:**
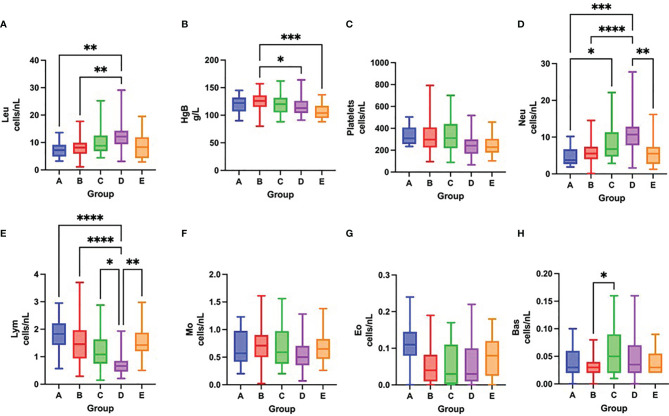
Differences in the total number of leukocytes **(A)**, concentration of hemoglobin **(B)**, total number of platelets **(C)**, neutrophils **(D)**, lymphocytes **(E)**, monocytes **(F)**, eosinophils **(G)** and basophils **(H)** among groups A – E after one week of hospitalization, Kruskal-Wallis test. *p < 0.05; **p < 0.01; ***p < 0.001; ****p < 0.0001.

On admission, COVID-19 severity correlated with leukocytosis, neutrophilia, lymphopenia, thrombocytopenia and eosinopenia (**Figures 3A–H** and **Table 3**). Over time, survival was accompanied by a significant increase in the total number of platelets and all leukocyte subsets, except for neutrophils. In contrast, fatal outcome was associated with a significant increase only in the number of platelets and neutrophil, eosinophil and basophil counts, while the total number of lymphocytes remained low during the first week of hospitalization (**Figures 4A–H** and **Table 3**).

**Table 3:** Results of differential blood cell counts and basic lymphocyte subsets in survivors (groups A – C) and non-survivors (group D) on admission to the hospital and after one week of hospitalization.

Parameter	Admission		After one week		S vs. NS		Changes over time	
							Admission vs. After one week	
	S	NS	S	NS	Admission	After one week	S	NS
	Median (IQR)	Median (IQR)	Median (IQR)	Median (IQR)	p value	p value	p value	p value
**Leukocytes [cells/nL]**	7.0 (5.1-9.3)	7.9 (5.4-11.3)	8.2 (6.0-10.2)	12.15 (9.4-14.28)	0.0015 **	<0.0001 ****	<0.0001 ****	<0.0001 ****
**Hemoglobin [g/L]**	130.0 (119.0-140.8)	122.0 (108.0-135.0)	124.5 (112.8-136.0)	113 (104-126)	<0.0001 ****	0.0111 *	0.0120 *	0.0014 **
**Platelets [cells/nL]**	231.5 (173.0-321.0)	179.0 (136.0-234.0)	305.5 (233.5-417.5)	242 (168.5-297.5)	<0.0001 ****	0.0029 **	<0.0001 ****	0.0153 *
**Neutrophils [cells/nL]**	5.28 (3.43-7.25)	6.25 (4.06-9.53)	5.62 (4,0-7.613)	10.72 (7.81-12.82)	<0.0001 ****	<0.0001 ****	0.4994 ns	0.0001 ***
**Lymphocytes [cells/nL]**	0.995 (0.67-1.40)	0.7 (0.45-1.03)	1.38 (0.91-1.895)	0.66 (0.46-0.85)	<0.0001 ****	<0.0001 ****	<0.0001 ****	0.9552 ns
**Monocytes [cells/nL]**	0.49 (0.350-0.698)	0.415 (0.278-0.620)	0.67 (0.47-0.92)	0.5 (0.35-0.705)	0.0004 ***	0.0151 *	<0.0001 ****	0.1042 ns
**Eosinophils [cells/nL]**	0.01 (0.0-0.02)	0 (0-0.02)	0.05 (0.01-0.11)	0.03 (0.01-0.1)	<0.0001 ****	0.4196 ns	<0.0001 ****	0.0005 ***
**Basophils [cells/nL]**	0.02 (0.01-0.03)	0.02 (0.01-0.03)	0.03 (0.02-0.05)	0.035 (0.02 – 0.07)	0.8838 ns	0.2308 ns	<0.0001 ****	0.0004 ***
**CD3^+^ [cells/uL]**	678 (424.5-984)	447 (271.3-678)	1024 (653.8 - 1447)	396 (289-697)	<0.0001 ****	<0.0001 ****	<0.0001 ****	0.4950 ns
**CD19^+^ [cells/uL]**	111 (64-177.5)	59 (30-109)	171 (91.25-269.8)	68.5 (55.5-164)	<0.0001 ****	0.0005 ***	<0.0001 ****	0.0150 *
**CD4^+^ [cells/uL]**	431.5 (257.8-625)	267 (154.5-414.5)	702 (418-1006)	269.5 (167.5-443.5)	<0.0001 ****	<0.0001 ****	<0.0001 ****	0.4223 ns
**CD8^+^ [cells/uL]**	178.5 (112.5-272)	118 (67-204.8)	231 (153-352)	115.5 (49.75-182.5)	<0.0001 ****	<0.0001 ****	<0.0001 ****	0.6083 ns
**IRI**	2.27 (1.56-3.28)	1.72 (1.14-2,89)	2.685 (1.923-3.828)	3.38 (1.638-4.873)	0.0001 ***	0.4664 ns	0.0010 **	0.1036 ns
**NK [cells/uL]**	145 (90-215)	125 (61-241)	136.5 (89.5-213.5)	76.5 (52.25- 140.5)	0.0399 *	0.0012 **	0.8671 ns	0.0090 **

Comparisons between survivors and non-survivors on admission and after one week of hospitalization (Mann-Whitney test), longitudinal changes in survivors and non-survivors (Wilcoxon rank-sum test). S, survivors; NS, non-survivors; IQR, interquartile range; ns, not significant, *p < 0.05; **p < 0.01; ***p < 0.001; ****p < 0.0001

**Figure 5:**
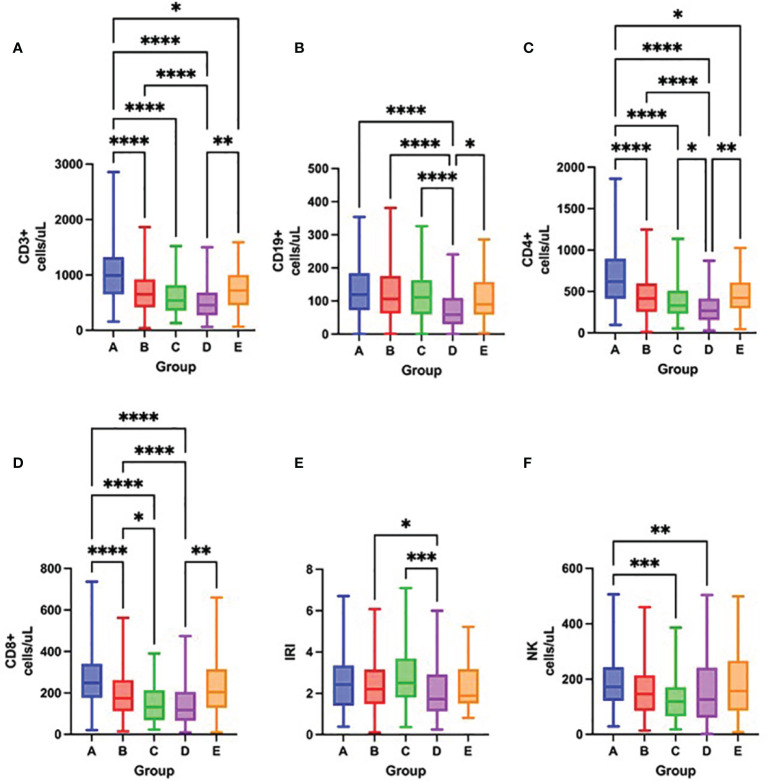
Differences in the total number of lymphocyte subpopulations measured by flow cytometry – CD3^+^ cells **(A)**, CD19^+^ cells **(B)**, CD4^+^ cells **(C)**, CD8^+^ cells **(D)**, IRI (immunoregulatory index CD4^+^/CD8^+^) **(E)**, NK cells **(F)** among groups A – E on admission to the hospital, Kruskal-Wallis test. *p < 0.05; **p < 0.01; ***p < 0.001; ****p < 0.0001.

**Figure 6:**
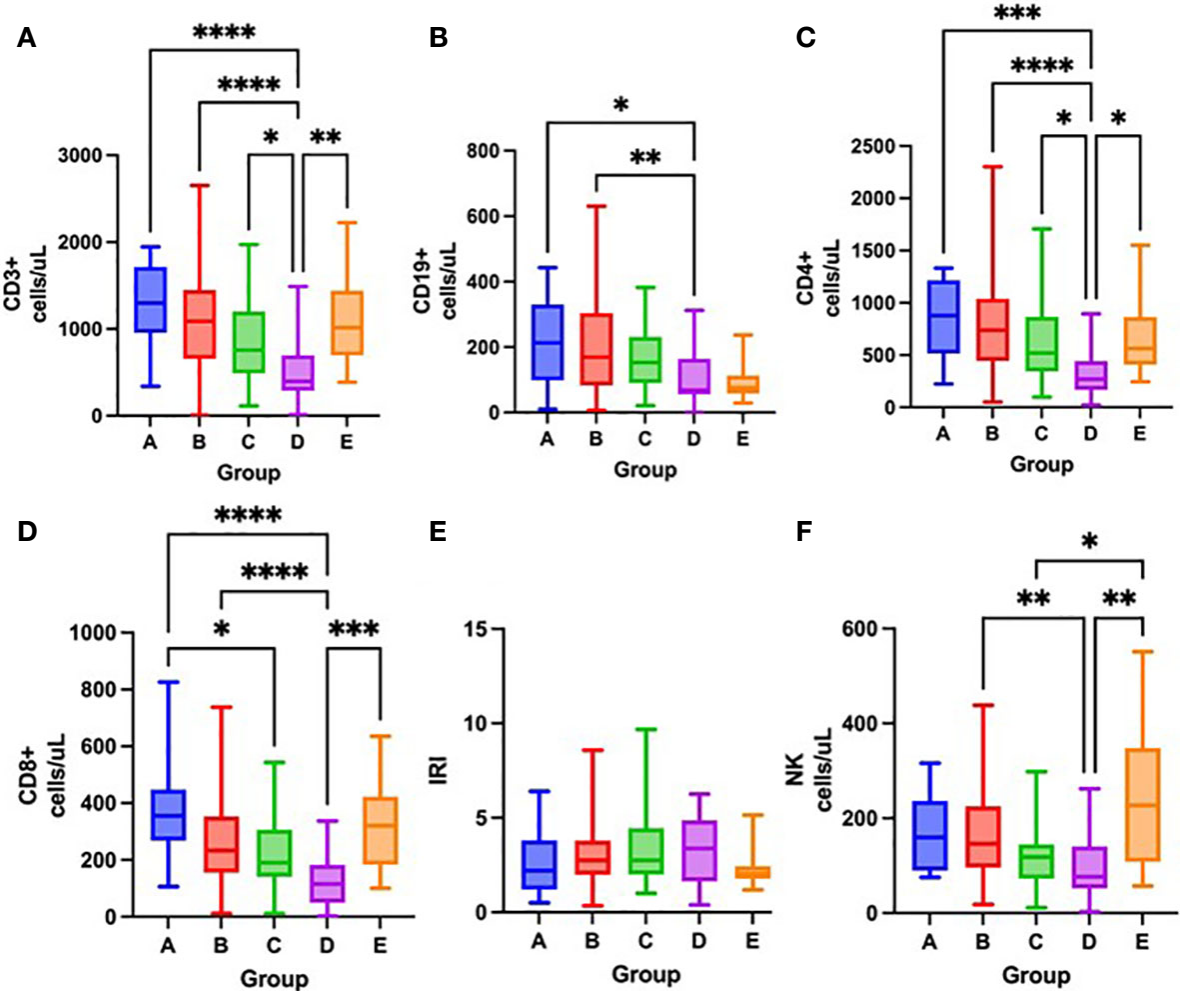
Differences in the total number of lymphocyte subpopulations measured by flow cytometry – CD3^+^ cells **(A)**, CD19^+^ cells **(B)**, CD4^+^ cells **(C)**, CD8^+^ cells **(D)**, IRI immunoregulatory index CD4^+^/CD8^+^) **(E)**, NK cells **(F)** among groups A – E after one week of hospitalization, Kruskal-Wallis test. *p < 0.05; **p < 0.01; ***p < 0.001; ****p < 0.0001.

Severity of lymphopenia on admission reflected in the depletion of all lymphocyte subsets, mostly CD3^+^, CD4^+^ and CD8^+^ cells (**Figures 5A–F** and **Table 3**). Over time, there was a significant increase in all lymphocyte subsets, except for NK cells in survivors. In contrast, only CD19^+^ cells increased in non-survivors, while NK cell counts further decreased (**Figures 6A–F** and **Table 3**).

### Non-Survivors Have Higher Proportion of CD8^+^CD38^+^ Cells and Lower Expression of CD159/NKG2A on CD8^+^ and NK Cells on Admission

On admission, as well as after one week of hospitalization, there were no significant differences in the expression of the activation marker HLA-DR on CD3^+^ cells nor in the co-expression of HLA-DR and CD38 on CD8^+^ cells between survivors and non-survivors. Similarly, no significant differences were observed in the proportion of CD4^+^CD45RO^+^ cells.

**Figure 7:**
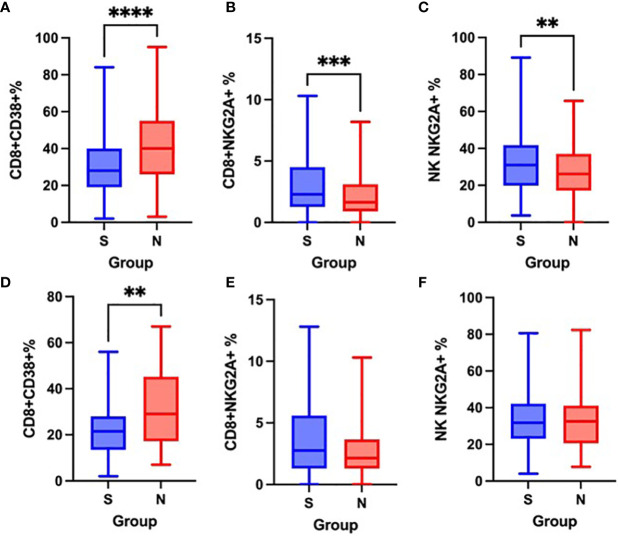
Differences in the proportion of CD8+CD38+ cells on admission **(A)** and after one week **(D)**, CD8^+^NKG2A^+^ cells on admission **(B)** and after one week **(E)** and NK NKG2A^+^ cells on admission **(C)** and after one week **(F)** between survivors [S] and non-survivors [N], Mann-Whitney test. **p < 0.01; ***p < 0.001; ****p < 0.0001.

Non-survivors had a significantly higher proportion of CD8^+^CD38^+^ cells on admission as well as after the first week of hospitalization (**Figures 7A, D**) and significantly lower expression of CD159/NKG2A on CD8^+^ and NK cells on admission, when compared to survivors (**Figures 7B, C**), however, no significant differences in CD159/NKG2A expression were seen after the first week (**Figures 7E, F**).

**Figure 8:**
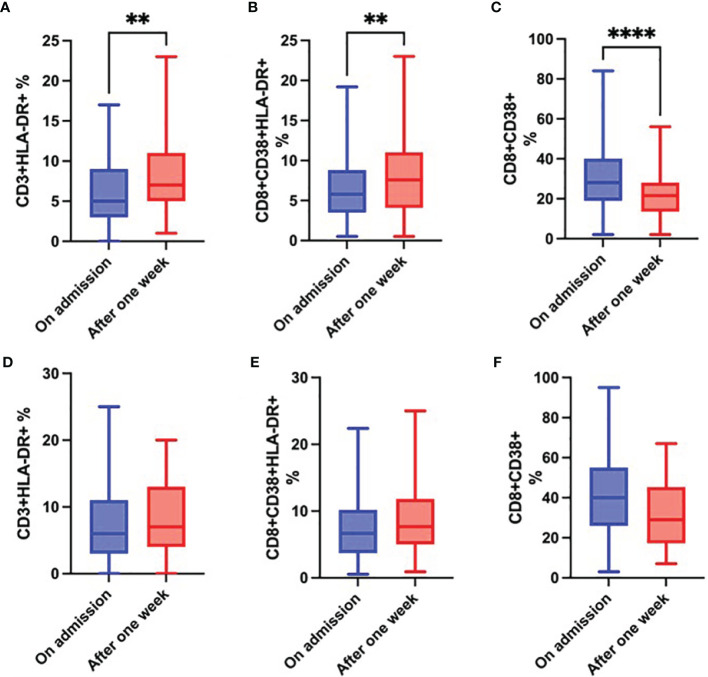
Changes over time in the proportion of CD3^+^HLA-DR^+^ cells **(A)**, CD8^+^CD38^+^HLA-DR^+^ cells **(B)** and CD8^+^CD38^+^ cells **(C)** in survivors; and changes over time in the proportion of CD3^+^HLA-DR^+^ cells **(D)**, CD8^+^CD38^+^HLA-DR^+^ cells **(E)** and CD8^+^CD38^+^ cells **(F)** in non-survivors during the first week of hospitalization, Wilcoxon rank-sum test. **p < 0.01; ****p < 0.0001.

While the proportion of CD3^+^HLA-DR^+^ and CD8^+^CD38^+^ HLA-DR^+^ cells (**Figures 8A, B**) significantly increased in survivors over time, the proportion of CD8^+^CD38^+^ cells significantly decreased (**Figure 8C**). Although changes in these parameters in the group of non-survivors followed the same trend, they were not significant (**Figures 8D–F**).

### Combinations of Selected Variables Have Better Prognostic Potential

We further investigated the prognostic potential of the examined parameters (survival versus death). To this end, we analysed ROC and calculated the AUC values for each parameter on admission and after one week of hospitalization. Except for several parameters measured after one week (the total number of lymphocytes, CD3^+^ and CD4^+^ cells), AUC values for other examined parameters were low both on admission as well as after one week (**Table 4**). Better results were achieved with combinations of selected variables (**Tables 5**, **6**).

**Table 4:** Area under the ROC curve (AUC) values for examined parameters on admission and after one week.

Parameter	AUC on admission	AUC after one week
**IgG [g/L]**	0.5412	–
**IgA [g/L]**	0.5015	–
**IgM [g/L]**	0.5809	–
**IgE [g/L]**	0.5412	–
**C3 [g/L]**	0.6190	–
**C4 [g/L]**	0.5153	–
**Leukocytes [cells/nL]**	0.5764	0.7242
**Hemoglobin [g/L]**	0.6156	0.6489
**Platelets [cells/nL]**	0.6738	0.6740
**Neutrophils [cells/nL]**	0.6099	0.7754
**Lymphocytes [cells/nL]**	0.6765	0.8097
**Eosinophils [cells/nL]**	0.6845	0.5488
**Basophils [cells/nL]**	0.5035	0.5720
**Monocytes [cells/nL]**	0.5862	0.6427
**CD3^+^ [cells/uL]**	0.6720	0.8091
**CD19^+^ [cells/uL]**	0.7090	0.7134
**CD4^+^ [cells/uL]**	0.6947	0.8100
**CD8^+^ [cells/uL]**	0.6336	0.7801
**IRI**	0.5946	0.5453
**NK [cells/uL]**	0.5501	0.6913
**CD3^+^HLA-DR^+^ [%]**	0.5383	0.5206
**CD8^+^CD38^+^ [%]**	0.6662	0.6748
**CD8^+^CD38^+^HLA-DR^+^[%]**	0.5481	0.5091
**CD8^+^NKG2A^+^ [%]**	0.5954	0.5795
**NK NKG2A^+^ [%]**	0.5819	0.5326
**CD4^+^CD45RO^+^ [%]**	0.5163	0.6008

**Table 5:** Area under the ROC curve (AUC) values for combination of selected variables on admission.

Selected combinations on admission	AUC
**C3** [g/L], **Hemoglobin** [g/L], **Platelets** [cells/nL], **Neutrophils** [cells/nL], **Lymphocytes** [cells/nL], **CD19^+^** [cells/uL], **CD4^+^** [cells/uL], **CD8^+^** [cells/uL], **CD8^+^CD38^+^** [%], **CD8^+^NKG2A^+^** [%], **NK NKG2A^+^** [%]	0.8755
**C3**[g/L], **Hemoglobin**[g/L], **Platelets** [cells/nL], **Lymphocytes** [cells/nL], **CD19^+^** [cells/uL], **CD4^+^** [cells/uL], **CD8^+^CD38^+^** [%]	0.8376
**Platelets**[cells/nL], **Neutrophils**[cells/nL], **Lymphocytes** [cells/nL], **CD3^+^** [cells/uL], **CD19^+^** [cells/uL], **CD4^+^** [cells/uL], **CD8^+^** [cells/uL], **CD8^+^CD38^+^** [%]	0.8154
**C3**[g/L], **Platelets** [cells/nL], **CD4^+^** [cells/uL], **CD8^+^CD38^+^** [%]	0.8088

**Table 6:** Area under the ROC curve (AUC) values for combination of selected variables after one week.

Selected combinations after one week	AUC
**Hemoglobin** [g/L], **Platelets** [cells/nL], **Neutrophils** [cells/nL], **Lymphocytes**[cells/nL], **CD3^+^** [cells/uL], **CD19^+^** [cells/uL], **CD4^+^** [cells/uL], **CD8^+^** [cells/uL], **CD8^+^CD38^+^** [%]	0.9127
**Neutrophils** [cells/nL], **CD4^+^** [cells/uL], **CD8^+^CD38^+^** [cells/uL], **CD8^+^NKG2A^+^** [%]	0.8765
**Platelets** [cells/nL], **Neutrophils** [cells/nL], **CD19^+^** [cells/uL], **CD4^+^** [cells/uL], **CD8^+^CD38^+^** [%]	0.8657
**Neutrophils** [cells/nL], **CD3^+^** [cells/uL], **CD8^+^CD38^+^** [%]	0.8611

## Discussion

In the present study, we focused on the analysis of the immune profile in hospitalized COVID-19 patients on admission and its changes over time. Except for already well described observations in the blood cell counts and basic lymphocyte subsets (11, 12, 15, 18, 21–35), we examined the expression of selected activation and inhibitory markers. We found a significantly lower expression of CD159/NKG2A on CD8^+^ and NK cells and a significantly higher expression of CD38 on CD8^+^ cells on admission in COVID-19 non-survivors. Over the first week of hospitalization in survivors, we observed a significant increase in HLA-DR expression on CD8^+^ and CD3^+^ cells and a significant decrease in the expression of CD38 on CD8^+^ cells. We did not find any prediction markers of fatal outcome.

Although COVID-19 predominantly affects the respiratory system, various other organs can be affected, associated and/or reflected in changes in humoral, immunological as well as hematological parameters. Leukocyte count abnormalities are commonly reported in COVID-19 patients (21, 24–30). Our findings concerning differential blood cell counts are in accordance with previously published data (11, 12, 15, 21–33). Severity of COVID-19 correlated mostly with the severity of thrombocytopenia, leukocytosis/neutrophilia, and lymphopenia. While eosinopenia is one of the laboratory hallmarks of COVID-19 infection (27), unlike our results, a pooled analysis (36) did not observe any difference in eosinophil count between severe and non-severe COVID-19 patients. Lymphopenia reflects in the depletion of lymphocyte subsets to various extent. The decrease in the total number of all lymphocyte subsets correlating with increasing severity of the disease is in line with other published results (34, 35).

Several authors suggested that COVID-19 is associated with dysregulated immune response. With respect to this, not only changes in the absolute count of lymphocyte subsets, but also differences in their functional status must be considered (11, 12, 15, 17). Dysregulated and uncoordinated innate immune response in older age (37, 38) might be associated with unsuccessful virus elimination in early stages of infection and subsequent excessive inflammation (15). Persistent excessive inflammatory responses with overactivation of lymphocyte subsets and subsequent cell exhaustion, anergy and apoptosis could explain the disease course in patients with critical disease (39). While increased expression of inhibitory molecules in cancer and chronic infection is referred as immune paralysis, their role in acute infection is still unclear and may potentially have both harmful and beneficial effects. Well-established balance between the expression of activation and inhibitory markers might be crucial (40). Mathew et al. (17) described prolonged T-cell activation during COVID-19 compared to other viral infections, what might reflect failing down-regulation of immune responses and possibly lead to cytokine storm.

NK cells and CD8^+^ cells, being responsible for killing virus-infected cells, represent an essential part of anti-viral immunity. If not appropriately regulated, their response can lead to the serious tissue damage. Several mechanisms, including expression of activation and inhibitory molecules, are responsible for such regulation (41).

NKG2A is a cell surface molecule expressed mostly by NK cells and activated CD8^+^ cells. As a heterodimer NKG2A/CD94, it binds to HLA-E and transduces inhibitory signals (42), its blockade was therefore considered in cancer treatment (43). Although inhibitory receptors in chronic viral infections are in general associated with T cell exhaustion and viral spreading (44), it was shown, that during an acute viral infection, NKG2A is necessary to counterbalance overactivation, prevent apoptosis, sustain the specific CD8^+^ cell response (45), and has tissue-protective effects (41).

CD38 and HLA-DR are, besides their other functions, associated with cell activation during immune response. The level of CD8^+^ cell activation depends on their combination (46). Co-expression of HLA-DR and CD38 in acute viral infection is associated with high proliferation, cytotoxicity and viral clearance (47). However, during chronic infection, this highly activated phenotype is later related to the loss of their functions, immune exhaustion and activation-induced cell death. In contrary, CD8^+^CD38^–^HLA-DR^+^ cells, despite their lower activation status, were associated with increased ability to suppress viral replication and overall better control in HIV patients (46). In relation to COVID-19, it was shown, that two different subpopulations of CD8^+^CD38^+^HLA-DR^+^ cells are present in COVID-19 patients. The subset of CD8^+^CD38^hi^HLA-DR^+^ T cells was considered overactivated with diminished effector function, prone to apoptosis, related to immune dysregulation, systemic inflammation and tissue injury in severe COVID-19 patients (48).

We found a significantly higher proportion of CD8^+^CD38^+^ cells in non-survivors compared to survivors both on admission and after one week. At the same time, proportions of CD8^+^NKG2A^+^ cells as well as NK NKG2A^+^ cells on admission were significantly lower in patients with fatal outcome. During hospitalization, we observed a significant increase in HLA-DR expression on both CD3^+^ and CD8^+^ cells in survivors, while the proportion of CD8^+^CD38^+^ cells in this group significantly decreased. Although such trend was also seen in non-survivors, differences were not significant. These results might point to unbalanced inhibition – activation status in patients with fatal outcome of COVID-19.

To date, little attention has been paid to CD159/NKG2A in relation to COVID-19. Increased expression of CD159/NKG2A in COVID-19 patients compared to healthy controls was reported by Zheng et al. (49), proportion of CD8^+^ and NK cells expressing CD159/NKG2A decreased during the disease course (49). Based on Zheng’s results (49), it was speculated that functional exhaustion of cytotoxic cells is responsible for impaired anti-viral response (50). Our results, pointing to better outcome of COVID-19 in individuals with higher expression of CD159/NKG2A on CD8^+^ and NK cells, support rather its protective role than functional exhaustion in acute viral infections.

The expression of various other inhibitory receptors on different lymphocyte subpopulations was studied in detail in COVID-19 patients (40). In general, upregulation of PD-1, TIM-3 and LAG-3 correlated with the disease severity in COVID-19 patients and was assigned to the lymphocyte exhaustion (11, 13, 39, 51–54). Less consistent evidence of association with COVID-19 disease severity is available for other inhibitory receptors TIGIT, BTLA, CTLA-4, VISTA and CD224 (52, 55–58). Importantly, increased expression of inhibitory receptors on lymphocytes in acute infection does not necessarily negatively affect their functionality and it also correlates with expression of activation markers (59, 60). The consequences of upregulation of inhibitory receptors may reflect compensatory counterbalance and should therefore be carefully considered in a complex way.

The possible role of CD38 in the pathogenesis of COVID-19 was recently highlighted by Horenstein et al. (61). CD38 has multiple functions. It induces secretion of various cytokines and regulates the migration of immune cells to the site of inflammation. In addition to operating as a signalling receptor and a marker of immune cell activation, CD38 possesses a nucleotidase enzymatic activity. The products of its enzymatic activity can contribute to the cytokine storm and lung immunopathology. It is also involved in cell adhesion and uncontrolled activation of immune cells could therefore contribute to lymphopenia and thrombosis (61).

Several studies examined T cell activation in the settings of various acute viral infections. In addition to the activation of virus-specific T cells, acute viral infections trigger the activation of T cells specific to persistent herpesvirus infection, that might contribute to both anti-viral immune response and virus associated immunopathology (62).

The peak of CD38 and HLA-DR expression corresponded to expected culmination of adaptive immune response during acute HBV, dengue and influenza infection (62). While increased T cell activation was reported in more severe disease (63, 64), the adenoviral infection was associated with only a slight increase in the activation of T cells (3.5%) compared to healthy controls (2.5%) (62). In contrast to mild influenza patients, T cell activation (expression of CD38 and HLA-DR) in severe influenza patients was delayed and/or exaggerated and associated with accumulation of partially differentiated cells suggesting disturbed migration of the effector cells to the site of infection (63).

Proportions of CD8^+^CD38^+^ HLA-DR^+^ and CD8^+^CD38^+^ cells were significantly increased in dengue fever patients compared to healthy controls. During the convalescent phase, CD8^+^CD38^+^HLA-DR^+^ cells, but not CD8^+^CD38^+^ cells, significantly decreased. Interestingly, despite maintaining their effector functions, impaired *in vitro* production of IFN- γ was detected and attributed to prevention of excessive inflammation (64).

Increased proportion of CD8^+^CD38^+^ cells on admission and after the first week of hospitalization in non-survivors in our study could potentially result from initial higher viral load. Both direct virus damage and exaggerated CD8^+^ activity can contribute to excessive tissue damage with further consequences. Thevarajan (65) described an increase in the co-expression of HLA-DR and CD38 on CD8^+^ T cells before clinical recovery in a patient with COVID-19 (65), what is in line with our findings of a significant increase in HLA-DR expression on CD3^+^ and CD8^+^ cells over the first week in survivors and suggests the importance of HLA-DR in reaching the control over acute viral infections. Conflicting results regarding the expression of activation markers were published by other authors (39, 66), what could possibly be explained by differences in compared groups, captured disease stage, therapeutic approaches, as well as various definitions of severity of the disease.

We observed significantly lower serum concentrations of IgM and C3 in non-survivors compared to survivors. Similarly to our previous study (11), there was a decreasing trend towards lower serum IgG concentration with increasing severity of COVID-19, deceased patients had surprisingly higher concentrations of IgG compared to critically ill patients. Lower concentration of IgM and significantly higher concentration of IgG and C3 in severe compared to non-severe cases were reported previously (15, 34).

Although severe COVID-19 elicited robust production of specific IgM and IgA antibodies in both COVID-19 survivors and non-survivors, decreased IgG response with impaired function of these antibodies was seen in non-survivors (67). In contrast, a meta-analysis found significantly higher specific IgG and IgA antibodies and slightly lower specific IgM in patients with severe COVID-19 (18). Disproportionate IgG subclass response with an increased binding to the inflammatory receptor FcγRIIIa was also reported (68).

Overactivation of the complement system, mainly C3a and C5a, participates in the pathophysiology of severe COVID-19 and is expected to contribute to the development of the cytokine storm, endothelitis as well as thromboembolic events (69). Patients, whose disease was associated with uncontrolled complement activation and consumption of C3, were more likely to die compared to patients with complement activation without consumption (70).

Although several reports of favorable outcome of COVID-19 in patients with primary antibody deficiency were published (71, 72), IgG-deficient patients presented with a more severe disease course and a higher risk of complications and death in a German study (73). Low IgG levels could be associated with an increased risk of nosocomial superinfections complicating disease course.

It was shown, that selected parameters of immune profile (total number of lymphocytes, CD4^+^, CD8^+^, CD19^+^) might be used as predictors of severe COVID-19, with AUC values > 0,75 (34, 74). In our study, none of examined parameters alone had sufficient sensitivity nor specificity to discriminate between survivors and non-survivors, the highest AUC values were obtained for lymphocyte and neutrophil counts and the total number of CD3^+^, CD4^+^ and CD8^+^ cells. However, unlike other studies (34, 74), we focused on discrimination between COVID-19 survivors and non-survivors, not severe and non-severe COVID-19 cases. As we have reported, patients with critical (group C) and fatal (group D) disease course did not differ significantly in most examined parameters. The lack of significant differences between these two groups let us speculate about possible space for therapeutic intervention.

Several applied therapeutic approaches could have an impact on observed immune signatures and the disease outcome. Although first samples were collected on admission, prior to administration of medications with such potential, samples after one week are expected to be affected. Moreover, due to evolving recommendations, some of therapeutic approaches changed over time. Regarding therapeutic strategy, major differences were seen when comparing groups A, E with groups B, C and D (**Table 2**). Although relatively high proportion of group A and E patients were treated with systemic corticosteroids, which are, in general, indicated for severe and critical COVID-19 (75), they were used for various different indications (*e.g.*asthma or COPD exacerbations) in these two groups.

Antivirals can decrease the viral load (76) and therefore impact the whole interaction of the virus and the immune system. Selected immunomodulators (*e.g* systemic corticosteroids, interleukin-1, interleukin-6 and JAK-inhibitors) were gradually added to the COVID-19 therapeutic repertoire and are used to mitigate excessive inflammatory responses associated with the disease progression (75). Among our patients, we mostly used dexamethasone and baricitinib.

In a French study, a low dose of dexamethasone in COVID-19 patients with ARDS was associated with more profound immune dysfunction on day 1 (lower expression of HLA-DR on monocytes and lower CD4^+^ cell counts) but also prevented fever and shortened the mechanical ventilation duration. Over the first week, the lymphocyte and CD4^+^ cell counts significantly increased in patients treated with dexamethasone. No significant increase in these cell counts and a significant decline in monocyte HLA-DR expression was confirmed in dexamethasone untreated group (77). Another transcriptomic preprint study analyzed the bronchoalveolar lavage fluid in ARDS patients with or without COVID-19, treated or untreated with dexamethasone. The use of dexamethasone in COVID-19 ARDS did not affect the expression of key pro-inflammatory genes, however interferon-stimulated genes were particularly upregulated in COVID-19 ARDS patients untreated with dexamethasone. Administration of dexamethasone in COVID-19 ARDS patients on the other hand lead to upregulation of genes related to B-cell and complement activation, antigen presentation, phygocytosis and FC-gamma receptor signalling (78).

The impact of baricitinib use on immune profile in COVID-19 patients provided Bronte et al. (79) Baricitinib restored the total number of circulating T and B cells and increased antibody production against the SARS-CoV-2 protein, but did not affect NK cells, neutrophils nor activated CD8^+^ cells (79).

However, our study was not designed to assess the influence of immunomodulator medication, which was administered to our patients at different time points during the hospitalization with respect to current guidelines, availability of the medication and the progression of the disease, regardless collection of the initial and control blood samples.

Among limitations of this study, we can mention the absence of the non-infected healthy control group, unbalanced group sizes, lacking group of non-hospitalized COVID-19 patients and the fact, that only two measurements (on admission and after one week) were included. Moreover, the time between the onset of the infection and hospital admission was variable, what could potentially impact results. It could be beneficial to correlate immune profile with viral load, however, but this information was not available for all patients.

COVID-19 pandemic allowed the scientists worldwide to study innate and acquired immune responses towards natural viral infection in details. Our results show that analysis of the immune profile on admission may be helpful in monitoring and prediction of the disease course in hospitalized COVID-19 patients. As none of examined parameters alone was able to predict the disease outcome with sufficient sensitivity and specificity, it is necessary to assess immune parameters in a more complex way, together with another clinical and laboratory predictors.

## Data Availability Statement

The raw data supporting the conclusions of this article will be made available by the authors, without undue reservation.

## Ethics Statement

The studies involving human participants were reviewed and approved by Ethical Committee of Martin University Hospital and Ethical Committee of Jessenius Faculty of Medicine, Comenius University in Bratislava. The patients/participants provided their written informed consent to participate in this study.

## Author Contributions

AB was involved in literature search, collection, analysis and interpretation of the data, drafting and revising the manuscript. MB was involved in flow cytometric analysis and analysis of the data. RV was involved in drafting, revising and editing the manuscript. JP was involved in flow cytometric analysis. IK was involved in revising and editing the manuscript. ZD was involved in revising and editing the manuscript. MJ was involved in literature search, analysis and interpretation of the data, drafting and revising the manuscript. All authors contributed to the article and approved the submitted version.

## Funding

This study has been produced with the support of the project KEGA 048UK-4/2021 and the Integrated Infrastructure Operational Program for the project: Creation of a Digital Biobank to support the systemic public research infrastructure, ITMS: 313011AFG4, co-financed by the European Regional Development Fund.

## Conflict of Interest

The authors declare that the research was conducted in the absence of any commercial or financial relationships that could be construed as a potential conflict of interest.

## Publisher’s Note

All claims expressed in this article are solely those of the authors and do not necessarily represent those of their affiliated organizations, or those of the publisher, the editors and the reviewers. Any product that may be evaluated in this article, or claim that may be made by its manufacturer, is not guaranteed or endorsed by the publisher.
